# Efficacy and Safety of CT‐Verified Deep Needling at the Foramen Acupoint for Lumbar Disc Herniation: A Randomized Controlled Trial

**DOI:** 10.1155/prm/9030734

**Published:** 2026-06-28

**Authors:** Wang Hao, Zhang Liang-hua, Chen Wei, Li Wei, Ning Zhen-zhen, Yu Xiao-gang, Bai Peng

**Affiliations:** ^1^ Beijing University of Chinese Medicine Third Affiliated Hospital, Beijing, China, bucm.edu.cn; ^2^ Department of Acupuncture and Moxibustion, Beijing Hospital of Integrated Traditional Chinese and Western Medicine, Beijing, China, bjcy2y.com; ^3^ School of Acupuncture-Moxibustion and Tuina, Beijing University of Chinese Medicine, Beijing, China, bucm.edu.cn

**Keywords:** acupuncture, computed tomography, deep needling, foramen acupoint, lumbar disc herniation, randomized controlled trial

## Abstract

**Objective:**

To evaluate the efficacy and safety of deep needling (DN) at the foramen acupoint compared to conventional needling (CN) and Huatuo Jiaji (HJ) acupuncture for lumbar disc herniation (LDH), needle depth was verified using computed tomography (CT).

**Methods:**

In this single‐center, randomized, three‐arm controlled trial, 165 participants with LDH were equally assigned to either the DN, CN, or HJ group. The DN group received needling at the foramen point to a depth of 60–70 mm, confirmed by CT to ensure it reached the outer intervertebral foramen. The CN group underwent routine‐depth needling, while the HJ group received standard HJ acupuncture. Treatments were administered three times a week for 4 weeks. Primary and secondary outcomes, including the Visual Analog Scale (VAS), Japanese Orthopaedic Association (JOA) scores, and Oswestry Disability Index (ODI), were assessed at baseline, 4 weeks, and 12 weeks.

**Results:**

The DN group demonstrated significantly greater improvements in VAS, JOA, and ODI scores compared to the CN and HJ groups at both 4 and 12 weeks (all *p* < 0.05). The total efficacy rate in the DN group was 94.44%, whereas it was 78.00% in both the CN and HJ groups at 4 weeks (*p* = 0.0306). CT imaging confirmed accurate needle placement in the foramen region with a mean depth of 5.91 ± 0.67 cm. No serious adverse events were reported.

**Conclusion:**

CT‐verified DN at the foramen acupoint is a safe and superior therapeutic strategy for LDH, emphasizing the importance of precise needling depth. This study offers a reproducible protocol for future research and clinical practice.

**Trial Registration:** Chinese Registry of Clinical Trials: ChiCTR2200061374

## 1. Background

Lumbar disc herniation (LDH) is a degenerative disease of the lumbar spine [1], characterized by annulus fibrosus rupture, leading to nucleus pulposus protrusion that compresses or irritates spinal nerves and nerve roots, resulting in symptoms such as low back pain, leg pain, and numbness [2]. LDH frequently exhibits a chronic and recurrent trajectory, substantially diminishing patients’ quality of life and presenting considerable challenges to achieving complete resolution [3]. Epidemiological studies report an incidence rate of 2%–3% [4], with approximately 95% of cases occurring at the L4‐5 and L5‐S1 levels [5, 6]. The increasing prevalence of LDH, influenced by factors such as an aging population and lifestyle modifications, significantly affects patients’ daily activities, occupational performance, and mental well‐being [7, 8].

Modern medicine primarily attributes LDH to intervertebral disc degeneration. As individuals age, the nucleus pulposus loses water content, which reduces elasticity, narrows the intervertebral space, and destabilizes the vertebral segment. Additionally, the avascular nature of the intervertebral disc limits its regenerative capacity. The posterior‐lateral annulus fibrosus is relatively weak, and at the L4‐S1 level, the posterior longitudinal ligament narrows, further compromising disc stability [9, 10]. These factors, combined with mechanical stressors such as excessive loading, trauma, or poor posture, can lead to disc herniation [10]. Current theories on pathogenesis include mechanical compression, chemical inflammation, and autoimmune responses [11, 12].

Treatment goals for LDH prioritize symptom relief, functional improvement, and prevention of recurrence. These treatment modalities are generally divided into surgical and nonsurgical approaches. While surgical options (such as open surgery, minimally invasive techniques, and fusion) may offer quicker initial relief, they tend to show comparable long‐term outcomes to nonsurgical management [13, 14]. Surgery also entails higher costs, stricter indications, and greater risks [15]. Given the generally favorable natural history of LDH, most patients benefit from nonsurgical treatments [16, 17], making them the first‐line option for cases without significant neurological deficits [18, 19].

Common nonsurgical pharmacotherapies include nonsteroidal anti‐inflammatory drugs (NSAIDs) and corticosteroids. NSAIDs are considered first‐line analgesics for back pain because they inhibit prostaglandin synthesis. However, their efficacy in treating sciatica is limited, and they also carry risks for gastrointestinal complications [20, 21]. Corticosteroids offer potent short‐term anti‐inflammatory effects but are not recommended for long‐term use due to systemic side effects and insufficient long‐term data [22].

Acupuncture, as a nonsurgical therapy, has unique advantages in treating LDH [23]. However, the proliferation of diverse techniques and acupoint selections, along with a lack of standardization, has hindered the widespread and reproducible application of these methods. Our previous clinical observations indicated that deep needling (DN) at the foramen area leads to superior symptom improvement in LDH. Of note, the intervertebral foramen contains not only the nerve root but also the dorsal root ganglion (DRG), radicular vessels, transforaminal ligaments, and sinuvertebral nerves, all of which may be affected by DN. We hypothesize that the superior efficacy of DN may involve multiple, potentially complementary mechanisms. First, direct stimulation of the compressed nerve root may trigger a reflexive withdrawal, disrupting adhesions and altering the nerve root–disc relationship. Second, needling may act on other foraminal structures—such as the DRG, vessels, or ligaments—modulating nociceptive signaling or local mechanical/neural responses. Third, mechanical stimulation may initiate anti‐inflammatory and neuroprotective changes within the foraminal microenvironment. This study aims to test these hypotheses and establish a reproducible protocol by employing computed tomography (CT)–guided three‐dimensional imaging. This approach will objectively verify the precise location of the needle tip when the *deqi* sensation is achieved, thereby correlating anatomical accuracy with clinical outcomes.

## 2. Materials and Methods

### 2.1. Study Design

This single‐center, randomized, three‐arm, controlled trial was conducted at the Acupuncture Department of Beijing Hospital of Integrated Traditional Chinese and Western Medicine. Patients with LDH were enrolled from July 2022 to July 2024. The study received ethical approval from the Regional Ethics Committee (No. ZXYEC‐KT‐2022‐01‐P01) and was registered with the Chinese Clinical Trial Registry. All participants provided written informed consent, and the study followed the principles outlined in the Declaration of Helsinki.

### 2.2. Participants

Inclusion criteria were as follows: (1) meeting the diagnostic criteria for LDH [24]; (2) age 25–65 years; (3) a leg pain Visual Analog Scale (VAS) score between 4 and 8; (4) symptom duration ≤ 8 weeks; and (5) provision of informed consent.

Exclusion criteria were as follows: (1) history of lumbar spine surgery or indications for surgery; (2) low back pain from other causes (e.g., fracture and tumor); (3) pregnancy or planned pregnancy; and (4) participation in other clinical trials within the preceding 3 months.

### 2.3. Randomization and Blinding

Eligible participants were randomly assigned in a 1:1:1 ratio to one of three intervention groups: the Huatuo Jiaji (HJ) group, the conventional needling (CN) group, or the DN group. An independent statistician, who had no involvement in participant recruitment, treatment, or assessment procedures, generated the randomization sequence using the random number generation function in SPSS software (Version 20). To ensure rigorous allocation concealment, this sequence was placed in sequentially numbered, opaque, sealed envelopes.

Upon enrollment, an independent research assistant, also uninvolved in treatment delivery or outcome assessment, opened the next consecutive envelope to reveal the group assignment to treating acupuncturist. However, this information was not disclosed to the participant, outcome assessors, or data analysts. Due to the inherently different nature of the acupuncture techniques, such as needle depth and target location, the acupuncturists could not be blinded to group assignment. All acupuncturists were instructed to refrain from discussing treatment details with participants or assessors.

Outcome assessors and data analysts remained blinded throughout the trial. Assessors responsible for data collection at baseline, 4 weeks, and 12 weeks were independent of the treatment team and had no access to randomization records or clinical notes. Participants were reminded not to disclose treatment details during assessments. The statistician conducting the final analysis remained blinded to group identity until of all primary statistical comparisons.

### 2.4. Intervention

The acupoints were localized in line with the World Health Organization Standard Acupuncture Point Locations, with the following specific definitions:

 Foramen acupoint: Located 1.0 cun lateral to the lower border of the spinous process of the corresponding responsible lumbar vertebra (L4, L5, or S1).
 HJ (EX‐B2): Located 0.5 cun lateral to the lower border of the spinous process of the corresponding lumbar vertebra.
 Other acupoints: Standard locations for Huantiao (GB30), Fengshi (GB31), Yanglingquan (GB34), Xuanzhong (GB39), Zhibian (BL36), Weizhong (BL54), Chengshan (BL57), and Kunlun (BL60).

All acupuncturists involved in the study were licensed practitioners with a minimum of 5 years of clinical experience and underwent standardized training on the trial protocol. Disposable sterile stainless‐steel needles (Hucheng, Beijing Keyuanda Medical Supplies Factory, China) were employed for the procedures. The quantified needling parameters for all acupoints, with the exception of the foramen acupoint in the DN group, are detailed in Table 1. For all acupoints, except where otherwise specified, the “even reinforcing‐reducing method” was implemented, which is characterized by bidirectional needle rotation within an amplitude of 120°–180° at a rate of 60–120 rotations per minute, conducted until *deqi* was achieved.

**TABLE 1:** Standardized acupuncture manipulation parameters for common acupoint.

Acupoint	Insertion angle	Insertion depth	*Deqi* sensation required
Huatuo Jiaji (EX‐B2)	Perpendicular	20–30 mm (0.8–1.2 cun)	Yes
Huantiao (GB30)	Perpendicular	40–50 mm (1.5–2.0 cun)	Yes
Fengshi (GB31)	Perpendicular	25–40 mm (1.0–1.5 cun)	Yes
Yanglingquan (GB34)	Perpendicular	25–40 mm (1.0–1.5 cun)	Yes
Xuanzhong (GB39)	Perpendicular	15–20 mm (0.5–0.8 cun)	Yes
Zhibian (BL36)	Perpendicular	40–50 mm (1.5–2.0 cun)	Yes
Weizhong (BL54)	Perpendicular	25–40 mm (1.0–1.5 cun)	Yes
Chengshan (BL57)	Perpendicular	25–40 mm (1.0–1.5 cun)	Yes
Kunlun (BL60)	Perpendicular	15–20 mm (0.5–0.8 cun)	Yes

For participants with radiating leg pain, the following adjunct acupoints were added:

 Lateral lower extremity pain: Huantiao (GB30), Fengshi (GB31), Yanglingquan (GB34), and Xuanzhong (GB39) on the affected side.
 Posterior lower extremity pain: Zhibian (BL54), Weizhong (BL40), Chengshan (BL57), and Kunlun (BL60) on the affected side.
 Combined lateral and posterior pain: The acupuncturist selected five points from the eight listed above.

### 2.5. DN Group

Foramen acupoint: A 0.30‐mm × 75.0‐mm needle was administered perpendicular to a depth of 60–70 mm. Successful needling was defined by elicitation of a mild, transient sensation radiating to the affected lower limb. Other acupoints were needled according to Table 1 parameters.

### 2.6. CN Group

Foramen acupoint: A 0.30‐mm × 40.0‐mm needle was inserted perpendicularly to a depth of 20–30 mm, with the objective local *deqi* (soreness and numbness surrounding the needle). Huantiao (GB30): A 0.30 mm × 75.0 mm needle was inserted perpendicularly to a depth of 50–65 mm, with the primary endpoint being the elicitation of a mild, transient sensation radiating to the distal portion of the affected lower limb. Other acupoints were needled in accordance with the specifications outlined in Table 1.

### 2.7. HJ Group

Obligatory points: HJ (EX‐B2) points on the affected segment were needled as per Table 1. Huantiao (GB30) was needled identically to the CN group. Other acupoints were selected and needled according to Table 1 parameters.

In all groups, needles were retained for 30 min. Treatments were administered three times per week (with at least one day between sessions) over 4 weeks, totaling 12 sessions.

### 2.8. Outcome Measures

#### 2.8.1. Primary Outcome Measure

The VAS [25] assessed patient low back pain intensity (0–10), with lower scores indicating less pain.

#### 2.8.2. Secondary Outcome Measure

Lumbar spine function was assessed using the Japanese Orthopaedic Association (JOA) assessment scale [26] that contains subjective symptoms (9 points), physical signs (6 points), and daily activity capability (14 points), totaling 29 points (lower scores indicate poorer function). The Oswestry Disability Index (ODI) [27] assessed low back pain and lumbar functionality. It contains 10 sections scored 0–5 each, with higher scores denoting more severe dysfunction.

Assessments were performed at 3 time points: pretreatment (baseline), posttreatment (4 weeks after the initial assessment), and follow‐up (12 weeks after the initial assessment).

The recovery rate was calculated according to the following formula [28]: recovery rate (%) =  (posttreatment JOA − pretreatment JOA)/(29 [full score] − pretreatment JOA) × 100. Cure: ≥ 75%, markedly effective: 50%–75%, effective: 25%–50%, and ineffective: < 25%.

Safety was assessed from the frequency of adverse events (AEs).

The first acupuncture treatment for all three groups took place in the CT room. While the needles were retained, a Siemens SOMATOM Emotion 16‐slice spiral CT scanner was utilized to scan the lumbar spine segments. The vertebral body was scanned with a slice thickness of 5 mm and an interval of 5 mm. In contrast, the intervertebral disc was scanned with a slice thickness of 1 mm and an interval of 1 mm. The CT images were selected and measured directly using the imaging system at Beijing Integrated Traditional Chinese and Western Medicine Hospital.

### 2.9. Sample Size Calculation

According to the previous trials and relevant literature [29], the posttreatment VAS values were estimated as follows: DN group—1.71 ± 1.89, HJ group—2.96 ± 2.22, and CN—group 4.39 ± 2.63. Using two‐sided tests with *α* = 0.05 and power (1‐β) = 0.8 (PASS Version 11.0), pairwise comparison indicated about 46 participants per group. To accommodate for a 20% dropout rate, 55 participants per group were required, totaling 165 in a 1:1:1 ratio.

### 2.10. Statistical Analyses

Data analysis was performed according to the intention‐to‐treat principle, utilizing SPSS (Version 20.0). Normality was evaluated using the Shapiro–Wilk test, while homogeneity of variances was assessed with Levene’s test. Continuous variables are reported as mean ± SD for those with normal distribution and as median for those with nonnormal distribution; categorical variables are presented as frequencies (percentages). Between‐group comparisons of VAS, JOA, and ODI at posttreatment and follow‐up were performed using analysis of covariance (ANCOVA), with baseline scores included as covariates. In cases where ANCOVA assumptions were not med, the Kruskal–Wallis H test was employed. Post hoc pairwise comparisons were carried out using the LSD test with Bonferroni correction when appropriate. Within‐group changes were analyzed using either paired *t*‐tests or Wilcoxon signed‐rank tests. For categorical data, comparisons were made using chi‐square tests or Fisher’s exact test.

## 3. Result

### 3.1. Baseline Characteristics

**FIGURE 1:**
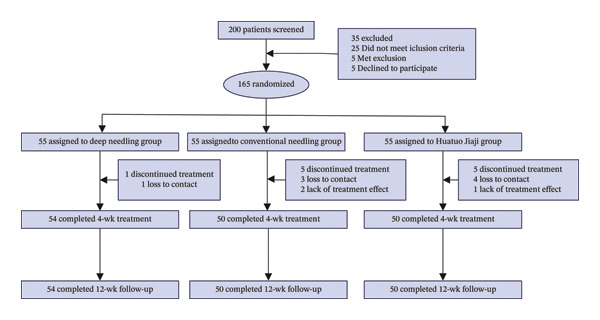
Participant flow diagram.

Of the 200 participants initially recruited, 165 were enrolled and randomized into the DN, CN, or HJ groups (Figure 1). The baseline demographic and clinical characteristics are summarized in Table 2.

**TABLE 2:** Baseline demographic and clinical characteristics of study participants.

Characteristic	No. (%)					
	Total	CN group	DN group	HJ group	Statistics	*p* value
Participants, No.	154	50	54	50		
Sex					0.58 (*x* ^2^)	0.7499
Male *N* (%)	66 (42.86)	22 (44.00)	21 (38.89)	23 (46.00)		
Female *N* (%)	88 (57.14)	28 (56.00)	33 (61.11)	27 (54.00)		
Age (year)					1.02 (*x* ^2^)	0.5995
Mean ± SD	49.70 ± 10.95	49.20 ± 11.35	51.24 ± 9.14	48.54 ± 12.29		
Median (IQR)	50.50 (19.00)	50.00 (19.00)	50.50 (15.00)	52.50 (23.00)		
Q1, Q3	41.00, 60.00	41.00, 60.00	45.00, 60.00	37.00, 60.00		
Min, Max	25.00, 68.00	25.00, 65.00	30.00, 65.00	26.00, 68.00		
Weight (kg)					0.65 (*x* ^2^)	0.7230
Mean ± SD	66.69 ± 11.86	67.82 ± 12.02	66.04 ± 11.58	66.26 ± 12.16		
Median (IQR)	65.00 (19.00)	67.00 (20.00)	65.00 (18.00)	67.00 (19.00)		
Q1, Q3	56.00, 75.00	56.00, 76.00	57.00, 75.00	56.00, 75.00		
Min, Max	42.00, 100.00	50.00, 94.00	50.00, 100.00	42.00, 88.00		
BMI (kg/m^2^)					0.04 (*x* ^2^)	0.9820
Mean ± SD	23.87 ± 3.41	23.86 ± 3.31	23.94 ± 3.26	23.80 ± 3.74		
Median (IQR)	24.00 (4.00)	24.00 (3.00)	24.00 (4.00)	24.00 (4.00)		
Q1, Q3	22.00, 26.00	22.00, 25.00	22.00, 26.00	22.00, 26.00		
Min, Max	16.00, 36.00	18.00, 35.00	19.00, 35.00	16.00, 36.00		
Duration of disease (year)					4.37 (*x* ^2^)	0.1123
Mean ± SD	0.92 ± 0.53	0.79 ± 0.45	1.03 ± 0.57	0.94 ± 0.55		
Median (IQR)	1.00 (0.50)	1.00 (0.50)	1.00 (0.50)	1.00 (0.50)		
Q1, Q3	0.50, 1.00	0.50, 1.00	0.50, 1.00	0.50, 1.00		
Min, Max	0.10, 2.00	0.10, 2.00	0.25, 2.00	0.30, 2.00		

*Note:* Participants consisted of 66 males and 88 females. The mean age was 51.24 ± 9.14 years in the DN group, 48.54 ± 12.29 years in the HJ group, and 49.20 ± 11.35 years in the CN group. The mean body mass index (BMI) was 23.94 ± 3.26 kg/m^2^ for the DN group, 23.80 ± 3.74 kg/m^2^ for the HJ group, and 23.86 ± 3.31 kg/m^2^ for the CN group. The average disease duration was 1.03 ± 0.57 months in the DN group, 0.94 ± 0.55 months in the HJ group, and 0.79 ± 0.45 months in the CN group.

### 3.2. VAS

**FIGURE 2:**
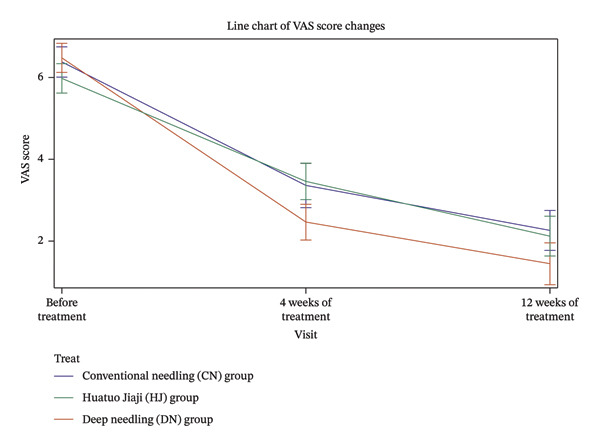
Line chart of VAS score changes.

No significant differences in baseline VAS scores were observed among the groups (DN: 6.48 ± 1.30; HJ: 5.98 ± 1.27; CN: 6.38 ± 1.31; *p* = 0.116), suggesting that initial pain levels were comparable. After 4 weeks, all groups showed significant reductions in scores (DN: 2.46 ± 1.60; HJ: 3.46 ± 1.57; CN: 3.36 ± 1.90; *p* = 0.0034), with each group’s improvements from baseline being statistically significant (*p* < 0.05). By 12 weeks, scores further decreased to 1.44 ± 1.87 for DN, 2.12 ± 1.71 for HJ, and 2.26 ± 1.72 for CN (*p* = 0.0116). The DN group demonstrated significantly greater improvement than both of the other groups at each time point (*p* < 0.05), indicating a stronger and more sustained analgesic effect associated with the DN intervention (Figure 2).

### 3.3. JOA Scores

**FIGURE 3:**
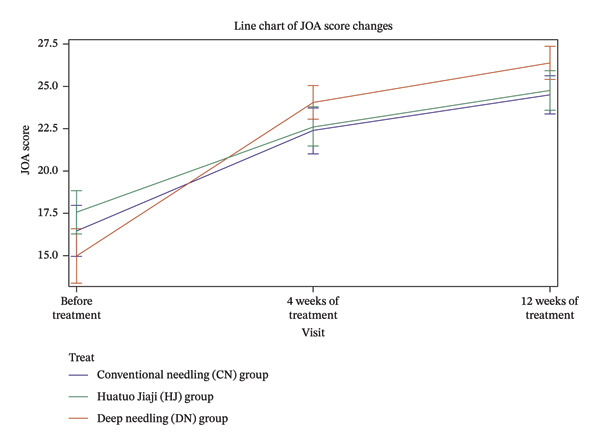
Line chart of JOA score changes.

Baseline JOA scores did not differ significantly among the three groups (DN: 14.98 ± 5.90; HJ: 17.56 ± 4.51; CN: 16.46 ± 5.31; *p* = 0.0879). However, due to the *p* value approaching 0.05, baseline‐adjusted analyses were utilized for all subsequent comparisons. After 4 weeks, scores increased significantly in all groups (DN: 24.06 ± 3.64; HJ: 22.60 ± 3.95; CN: 22.40 ± 4.90; *p* = 0.0002). These improvements were sustained at 12 weeks (DN: 26.39 ± 3.59; HJ: 24.76 ± 4.12; CN: 24.50 ± 3.97; *p* = 0.0012). The DN group exhibited the greatest improvement, followed by the CN and HJ groups, with intergroup differences in change from baseline being statistically significant at both time points (*p* < 0.05) (Figure 3).

### 3.4. ODI

**FIGURE 4:**
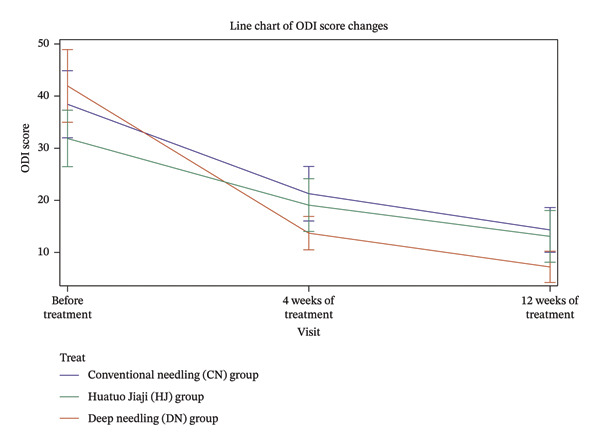
Line chart of ODI score changes.

Baseline ODI scores were comparable across the three groups (DN: 41.96 ± 25.58; HJ: 31.88 ± 19.05; CN: 38.44 ± 22.72; *p* = 0.1159). Significant reductions in scores were noted at 4 weeks (DN: 13.70 ± 11.72; HJ: 19.08 ± 17.79; CN: 21.28 ± 18.40; *p* = 0.0876) and at 12 weeks (DN: 7.22 ± 11.02; HJ: 13.08 ± 17.45; CN: 14.32 ± 15.09; *p* = 0.0017), with all groups showing statistically significant improvement from baseline (*p* < 0.05). The DN group achieved the most substantial reduction, followed by the CN and HJ groups. Intergroup differences in the magnitude of improvement were significant at both 4 and 12 weeks (*p* < 0.05) (Figure 4).

### 3.5. Overall Efficacy Based on JOA Scores

At 4 weeks, significant differences in clinical outcomes were observed among the groups (*p* = 0.0005). In the CN group, 12 patients (24.00%) were classified as cured, 15 (30.00%) as markedly effective, 12 (24.00%) as effective, and 11 (22.00%) as ineffective. The DN group showed a higher proportion of patients cured, with 19 (35.19%) cured, 25 (46.30%) classified as markedly effective, 7 (12.96%) as effective, and 3 (5.56%) as ineffective. In contrast, the HJ group had 5 cured (10.00%), 20 markedly effective (40.00%), 14 effective (28.00%), and 11 ineffective (22.00%).

By the 12‐week follow‐up, the intergroup differences remained significant (*p* = 0.0029). The CN group reported 19 cured (38.00%), 18 markedly effective (36.00%), 11 effective (22.00%), and 2 ineffective cases (4.00%). The DN group demonstrated the highest success, with 38 cured (70.37%), 8 markedly effective (14.81%), 6 effective (11.11%), and 2 ineffective patients (3.70%). The HJ group had 19 cured (38.00%), 19 markedly effective (38.00%), 6 effective (12.00%), and 6 ineffective (12.00%).

**FIGURE 5:**
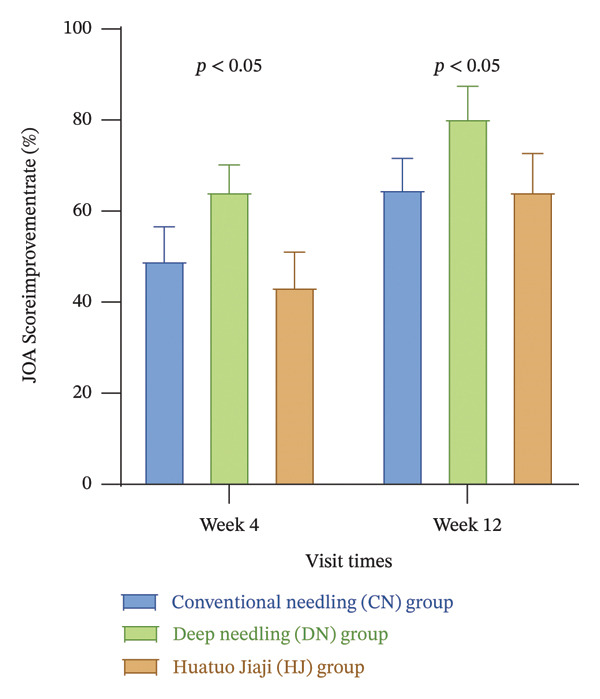
JOA score improvement rate (%).

The overall response rate at 4 weeks was 78.00% for the CN group, 94.44% for the DN group, and 78.00% for the HJ group (*p* = 0.0306). At 12 weeks, the overall response rates improved to 96.00% for CN, 96.30% for DN, and 88.00% for HJ (*p* = 0.2322) (Figure 5).

### 3.6. CT Image

**FIGURE 6:**
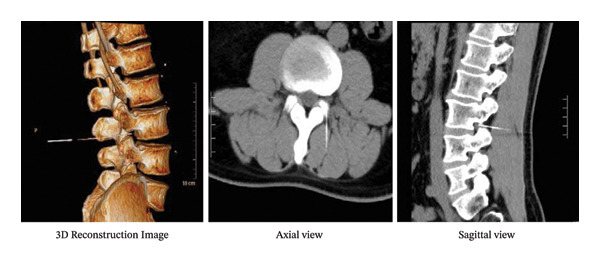
Lumbar CT image during deqi sensation of acupuncture in the DN group.

CT imaging conducted after achieving *deqi* in 54 patients from the DN group indicated that the needle tip consistently reached the outer orifice of the intervertebral foramen (Figure 6). The mean needle insertion depth was measured at 5.91 ± 0.67 cm.

Representative CT images for the CN and HJ groups are provided in Supporting Figure S1.

### 3.7. AEs

No serious AEs were reported in any of the groups (Table 3). In particular, there were no AEs that necessitated hospitalization or surgery, nor were there any related to the exacerbation of preexisting conditions.

**TABLE 3:** Incidence of treatment‐related adverse events.

Adverse events	No. (%)			
	Total	CN group	DN group	HJ group
Participants, No.	154	50	54	50
Hematoma/bruising	0	0	0	0
Dizziness	0	0	0	0
Minor bleeding	0	0	0	0
Post‐needling sensation (mild)	0	0	0	0

### 3.8. Concomitant Medication

No additional analgesics or physical therapies were permitted during the 4‐week treatment period. Rescue paracetamol use was minimal and did not differ significantly among the three groups (*p* > 0.05).

## 4. Discussion

The findings of this study indicate that CT‐verified DN at the foramen acupoint results in significantly greater and more sustained improvements in pain, function, and overall efficacy for LDH compared to CN and standard HJ (EX‐B2) acupuncture. Coupled with its favorable safety profile, these results suggest that the precision of needling depth and anatomical targeting are critical factors influencing the therapeutic outcomes of acupuncture for LDH.

A key methodological feature of this study is the objective verification of needle placement using CT imaging. This imaging confirmed that the needle tips in the DN group consistently reached the region of the outer intervertebral foramen, with a mean depth of 5.91 cm. This anatomical correlation offers a plausible explanation for the observed differences in efficacy. We propose that the therapeutic effects may be attributed to two interrelated mechanisms.

### 4.1. Potential Local Mechanical Neuromodulation

By positioning the needle close to the affected nerve root, DN might provide more direct mechanical stimulation. It is hypothesized that such stimulation could trigger a reflexive displacement of the nerve root, potentially altering its spatial relationship with surrounding adhesions or the herniated disc and thereby contributing to a degree of physical decompression. However, it must be emphasized that direct in vivo evidence supporting this “needle tip ⟶ nerve root displacement ⟶ decompression” chain is currently lacking. Preliminary ultrasound evidence indicates that needling can cause nerve displacement at both peripheral and intraspinal levels: needle contact with a peripheral nerve can induce lateral “rolling” displacement [30], while acupuncture treatment has been associated with posttreatment repositioning of the nerve root relative to the herniated disc in LDH patients [31], but this evidence is indirect. To date, no study has documented real‐time nerve root displacement induced by acupuncture using dynamic visualization during needling, musculoskeletal ultrasound, or neurophysiological monitoring. Therefore, the mechanical decompression hypothesis remains speculative. The radiating sensation (*deqi*) experienced in the distal lower limb, which our protocol defines as a requisite endpoint for successful needling, may serve as an indirect indicator of neural engagement but does not confirm mechanical displacement. Additionally, the needle tip may also be in close proximity to the DRG or radicular vessels. Stimulation of the DRG could modulate segmental nociceptive processing, as suggested by recent findings on HCN2 channel expression in the DRG after electroacupuncture [32]. Future investigations employing intraoperative nerve electrophysiology, musculoskeletal ultrasound, or fluorescent tracing in animal models are warranted to directly test this hypothesis.

### 4.2. Possible Modulation of the Local Pathological Microenvironment

The intervertebral foramen plays crucial role in inflammatory and immune responses related to LDH. By precisely targeting this area with needling, it may be possible to effectively modulate the local pathophysiological state. Recent studies have identified multiple molecular pathways through which acupuncture exerts anti‐inflammatory and neuroprotective effects in LDH models. Acupuncture suppresses proinflammatory cytokines (TNF‐α, IL‐1β, and IL‐6) and has been shown to modulate inflammatory mediator networks in LDH‐induced nerve injury [33]. The CXCL12/CXCR4 signaling axis has emerged as a critical mediator; acupuncture inhibits CXCL12/CXCR4‐driven glial activation and reduces ERK/NF‐κB phosphorylation, leading to neuropathic pain relief [34]. Clinical proteomic analysis further confirmed that CXCL12 and CXCR4 are among the core differentially expressed proteins in LDH patients that are reversed by acupuncture treatment, with expression levels significantly correlated with VAS, ODI, and JOA scores [35]. Emerging evidence also links needle‐based interventions to oxidative stress modulation and neuroprotection; electroacupuncture at Jiaji points upregulates the BDNF/NRF2 signaling pathway in rabbits with intervertebral disc degeneration, thereby reducing oxidative stress, protecting DRG neurons, and alleviating pain [36]. Collectively, these findings suggest that DN at the foramen acupoint may achieve therapeutic benefits through multimodal mechanisms, including cytokine suppression, glial modulation, and oxidative stress regulation. Future studies should incorporate serum cytokine profiling or MRI‐based biomarkers to directly examine these mechanisms in patients. Beyond these peripheral mechanisms, central nervous system modulation may also contribute. Recent fMRI evidence has shown that acupoint stimulation can alleviate LDH pain by regulating functional connectivity between the inferior frontal triangularis and multiple brain networks [37], suggesting that nonsurgical interventions may exert their effects partly through brain network reorganization.

Our study connects the technical parameter of “needling depth” to an imaging‐confirmed anatomical endpoint and correlates it with clinical outcomes, establishing a logical framework that explains the superior efficacy of DN.

These findings have significant clinical implications, advocating for a more standardized, anatomy‐informed approach to acupuncture for LDH. We justify the use of a specific needling depth (60–70 mm) at the foramen acupoint in patients with radicular symptoms, suggesting that precise and adequate stimulation within safe anatomical limits is crucial for optimal outcomes. Although CT guidance is not routinely used, the technique can enhance targeting precision safely when performed by trained practitioners using clear landmarks. This evidence supports the inclusion of anatomically‐grounded, depth‐standardized needling in nonsurgical management protocols for LDH, particularly in cases with definitive radicular involvement.

Compared with recent related studies [38, 39], this research demonstrates distinctive features in its methodological design and operational reporting. Current clinical acupuncture practice encompasses a variety of treatment protocols and variable point selection, frequently lacking standardized operational guidelines. This inconsistency may restrict reproducibility and broader application. To address these issues, our study implemented a structured intervention strategy by selecting the segmental foramen acupoint that corresponds to the patient’s clinical manifestations as the primary point, complemented by a limited number of channel‐based adjunct points. This methodology effectively balances clinical feasibility with procedural standardization. Furthermore, the introduction of CT imaging allows for the objective verification of needle placement in real time, thereby transforming needling depth from a subjective, experience‐based description into quantifiable objective data. This advancement enhances methodological rigor and offers a practical technical reference for future mechanistic investigations. Additionally, we systematically reported a series of quantified parameters—including needling angle, depth, manipulation technique, and *deqi* criteria—thereby establishing a transparent operational protocol. This contribution aims to rectify the often incomplete reporting of interventions and address the limited reproducibility observed in acupuncture clinical research.

### 4.3. Limitation

Certain limitations should be acknowledged. Firstly, the single‐center design may restrict the generalizability of the findings. To confirm these results, multicenter trials involving diverse populations are needed. Secondly, while patients and assessors were blinded, the acupuncturists could not be blinded because of the nature of the intervention. This limitation may introduce performance bias [40]. Future studies could benefit from utilizing sham acupuncture or nonpenetrating needles in control groups to more effectively isolate the specific effects associated with needling depth. Additionally, while the sample size was calculated a priori, it may still be inadequate to detect infrequent AEs or differences among subgroups.

Additionally, the study did not incorporate biochemical or imaging biomarkers to objectively quantify inflammatory or structural changes. The integration of MRI‐based assessments of disc morphology or serum cytokine levels could yield deeper insights into the mechanistic pathways involved. Long‐term follow‐up extending beyond 12 weeks would also be advantageous for evaluating the durability of treatment effects and recurrence rates.

Third, we did not prespecify or analyze outcomes based on LDH subtypes. In our clinical context, imaging findings often correlate poorly with symptom severity, and our primary focus was on patient‐centered outcomes (pain and function) rather than morphological classification. Nonetheless, the absence of subtype analysis limits our ability to determine whether the superiority of DN applies equally to all LDH types, especially calcified or sequestrated herniations. Future large‐scale trials with prospective stratification by herniation type and calcification status are warranted to address this question.

Despite these limitations, this study adds to the expanding body of evidence supporting the application of acupuncture in the management of LDH. It underscores the necessity of standardizing techniques within acupuncture research. The reproducible protocol detailed herein—including parameters such as needle depth, manipulation, and acupoint selection—provides a viable model for future clinical practice and research.

## 5. Conclusion

In conclusion, DN at the foramen acupoint appears to be a safe, effective, and durable treatment option for patients with LDH. Its demonstrated superiority over routine needling and Jiaji acupuncture indicates that both the depth and location of needling are critical factors influencing therapeutic outcomes. These findings advocate for the incorporation of DN techniques into clinical guidelines for the nonsurgical management of LDH, especially in cases where conventional acupuncture or pharmacotherapy has proven inadequate.

Future research should concentrate on elucidating the neurophysiological and immunomodulatory mechanisms underlying DN, optimizing treatment protocols, and validating these findings in larger, multicenter cohorts.

## Funding

This study was supported by grants from the Capital’s Funds for Health Improvement and Research (CFH‐4–7044).

## Conflicts of Interest

The authors declare no conflicts of interest.

## Supporting Information

Additional supporting information can be found online in the Supporting Information section.

## Supporting information


**Supporting Information** Supporting Figure S1. Representative CT images of needle placement in the CN and HJ groups. (a, b) CN group: axial (a) and sagittal (b) views showing the needle tip (white arrow) located within the paraspinal muscle at a depth of approximately 25 mm, not reaching the intervertebral foramen. (c, d) HJ group: axial (c) and sagittal (d) views showing the needle tip at the Huatuo Jiaji point (EX‐B2), positioned 0.5 cun lateral to the spinous process, at a depth of approximately 25 mm.

## Data Availability

The data that support the findings of this study are available on request from the corresponding author. The data are not publicly available due to privacy or ethical restrictions.
