# Supplementary material for: Efficacy and Safety of CT‐Verified Deep Needling at the Foramen Acupoint for Lumbar Disc Herniation: A Randomized Controlled Trial
Source: Pain Res Manag. 2026 Jun 28;2026:9030734. doi: 10.1155/prm/9030734 (PMC13310422; doi:10.1155/prm/9030734)
Supplement: Supplementary file 1 — Supporting Information Supporting Figure S1. Representative CT images of needle placement in the CN and HJ groups. (a, b) CN group: axial (a) and sagittal (b) views showing the needle tip (white arrow) located within the paraspinal muscle at a depth of approximately 25 mm, not reaching the intervertebral foramen. (c, d) HJ group: axial (c) and sagittal (d) views showing the needle tip at the Huatuo Jiaji point (EX‐B2), positioned 0.5 cun lateral to the spinous process, at a depth of approximately 25 mm. [file PRM-2026-9030734-s001.docx]

**Supplementary Figure S1. Representative CT images of needle placement in the CN and HJ groups.**

| 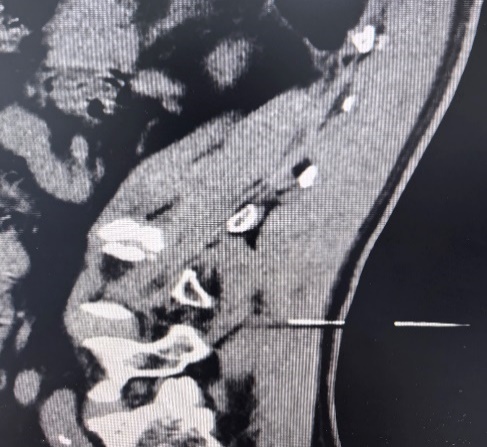**Lumbar CT Image in CN Group** | |
| --- | --- |
| 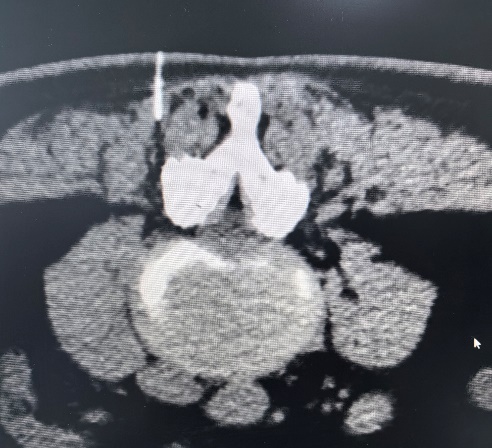 |  |
| **a. Axial View** | **b. Sagittal View** |

**(a)** CN group, axial view. The needle tip (white arrow) is located within the paraspinal muscle, approximately 25 mm from the skin surface, not reaching the intervertebral foramen. **(b)** CN group, sagittal view. Needle tip (arrow) in the same location.

| 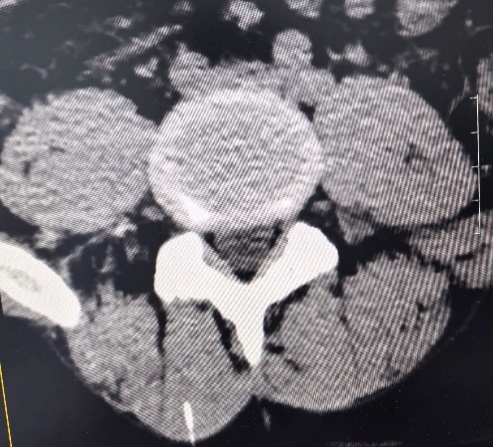**Lumbar CT Image in HJ Group** | |
| --- | --- |
|  | 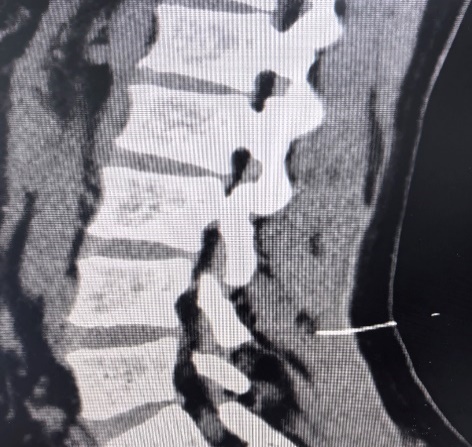 |
| **c. Axial View** | **d. Sagittal View** |

**(c)** HJ group, axial view. Needle tip (white arrow) at the Huatuo Jiaji point (approximately 25 mm depth), **(d)** HJ group, sagittal view.
